# Role of Chemokines in Thyroid Cancer Microenvironment: Is CXCL8 the Main Player?

**DOI:** 10.3389/fendo.2018.00314

**Published:** 2018-06-21

**Authors:** Mario Rotondi, Francesca Coperchini, Francesco Latrofa, Luca Chiovato

**Affiliations:** ^1^Unit of Internal Medicine and Endocrinology, ICS Maugeri I.R.C.C.S., Laboratory for Endocrine Disruptors, University of Pavia, Pavia, Italy; ^2^Department of Clinical and Experimental Medicine, University of Pisa, Pisa, Italy

**Keywords:** CXCL8, chemokines, thyroid, cancer, tumor-related inflammation, tumor microenvironment

## Abstract

Tumor-related inflammation does influence the biological behavior of neoplastic cells and ultimately the patient’s outcome. With specific regard to thyroid cancer, the issue of tumor-associated inflammation has been extensively studied and recently reviewed. However, the role of chemokines, which play a crucial role in determining the immuno-phenotype of tumor-related inflammation, was not addressed in previous reviews on the topic. Experimental evidence shows that thyroid cancer cells actively secrete a wide spectrum of chemokines and, at least for some of them, solid scientific data support a role for these immune-active molecules in the aggressive behavior of the tumor. Our proposal for a review article on chemokines and thyroid cancer stems from the notion that chemokines, besides having the ability to attract and maintain immune cells at the tumor site, also produce several pro-tumorigenic actions, which include proangiogenetic, cytoproliferative, and pro-metastatic effects. Studies taking into account the role of CCL15, C–X–C motif ligand 12, CXCL16, CXCL1, CCL20, and CCL2 in the context of thyroid cancer will be reviewed with particular emphasis on CXCL8. The reason for focusing on CXCL8 is that this chemokine is the most studied one in human malignancies, displaying multifaceted pro-tumorigenic effects. These include enhancement of tumor cells growth, metastatization, and angiogenesis overall contributing to the progression of several cancers including thyroid cancer. We aim at reviewing current knowledge on the (i) ability of both normal and tumor thyroid cells to secrete CXCL8; (ii) direct/indirect pro-tumorigenic effects of CXCL8 demonstrated by *in vitro* and *in vivo* studies specifically performed on thyroid cancer cells; and (iii) pharmacologic strategies proven to be effective for lowering CXCL8 secretion and/or its effects on thyroid cancer cells.

## Tumor-Related Inflammation

Inflammation is a physiologic protective process used by the organism in response to tissue damage. The strong relationship between inflammation and the development, maintenance and progression of cancer was hypothesized for the first time in the far 1863 (1) by Virchow. His observation that leukocytes infiltrate neoplastic tissues provided evidence for a connection between inflammation and cancer (2). Subsequently, inflammation was more specifically related to tumorigenesis because of its ability to favor genome instability, tumor cell growth, and angiogenesis. Accumulation of immune cells was also shown to influence tissue homeostasis. Thus, inflammation is currently regarded as an essential component of malignancies (3).

Further advances led to the identification of “inflammatory hallmarks” of cancer, which include inflammatory cells, inflammatory mediators, and factors involved in tissue remodeling–repair and angiogenesis. It is important to note that inflammatory features were also found in those tumors for which a firm causal relationship with inflammation was not established (4). As a consequence, the original notion that “inflammation causes cancer” was progressively changed to “not only inflammation can cause cancer, but also cancer can cause inflammation” (5). The above statement mainly relies upon the demonstration that oncogenic changes specifically promote the *de novo* production of inflammatory mediators (5). Further evidence supporting this novel view of the events derives from the observation that cancer cells can “manipulate” their microenvironment to escape immune surveillance. While inflammatory cells may recognize tumor cells as antigens, the tumor microenvironment itself is a source of factors that suppress antitumor immune responses (6).

Chemokines represent a crucial component of the network of inflammatory mediators associated with cancer and are by no doubts the most extensively characterized molecules involved in the maintenance and progression of tumor-related inflammation (7).

## Chemokines and Tumor Microenvironment

**Figure 1:**
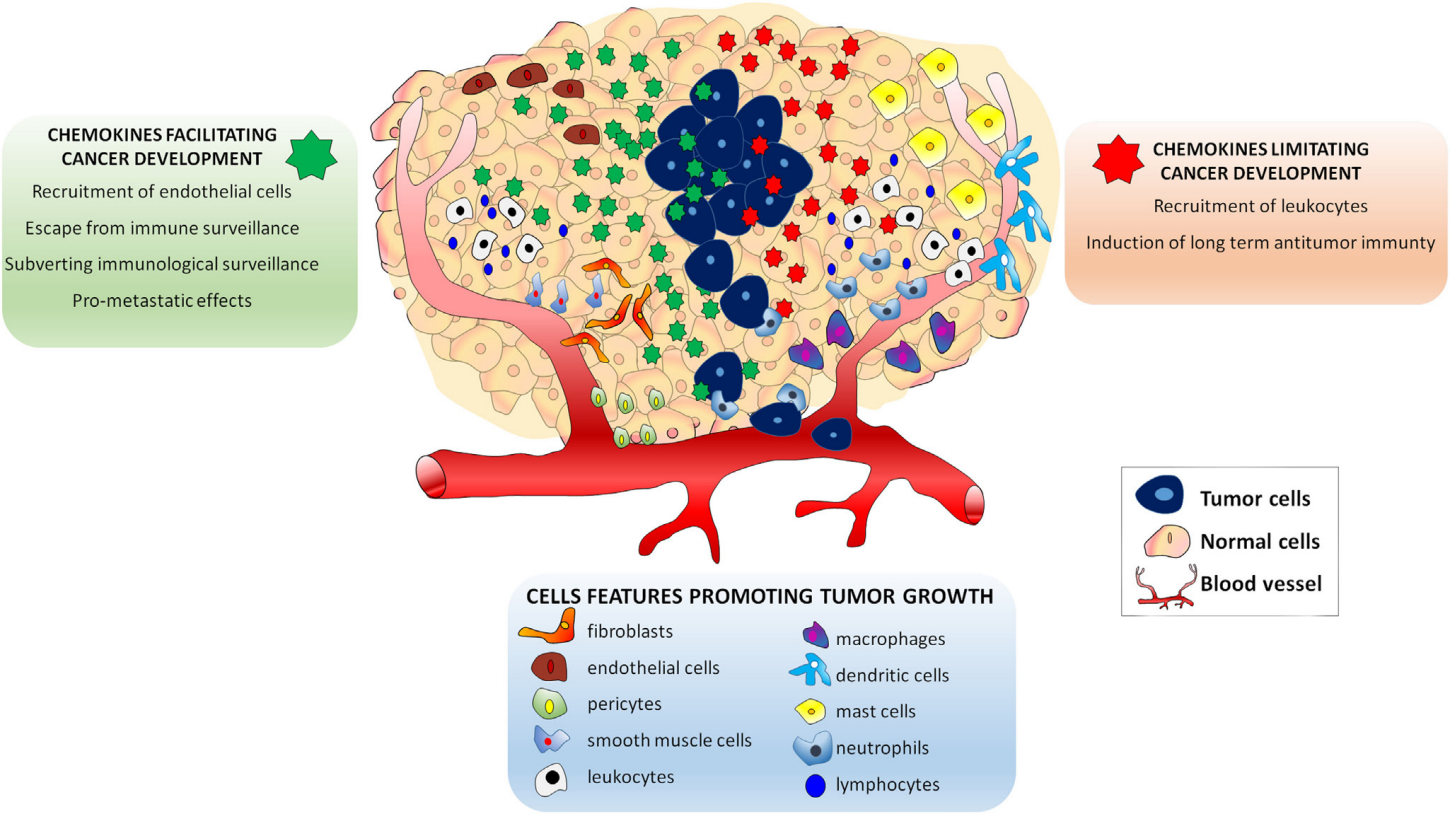
Effects of chemokines in the Tumor microenvironment. The presence of endothelial cells, pericytes and smooth muscle cells lymphocytes, macrophages, dendritic cells, mast cells, and neutrophils attracted by specific chemokines influences tumor growth. Chemokines secreted in tumor microenvironment play a dual role in the oncogenic process. Some chemokines (*green*) may facilitate tumor cell growth by: (i) recruiting endothelial cells; (ii) subverting immunologic surveillance; (iii) maneuvering the tumor leukocyte profile to escape antitumor immune surveillance, and (iv) inducing metastatic process. Other chemokines may limit neoplastic development (*red*) by: (i) increasing leukocyte migration and (ii) inducing long-term antitumor immunity.

Several studies in cancer patients showed that the inflammatory profile of tumor microenvironment is closely related to its biological behavior (8–10). Moreover, growing evidence recently reviewed, suggested that the composition of tumor microenvironment affects the therapeutic outcome of the patient (11, 12). The tumor microenvironment is made of extracellular matrix and stromal cells, which include fibroblasts, vessel cells (endothelial cells, pericytes, and smooth muscle cells), and inflammatory leukocytes [lymphocytes, macrophages, dendritic cells (DCs), mast cells, and neutrophils] (13). These cells, either alone or in combination, potentially contribute to tumor growth, being their recruitment regulated by the presence of specific chemokines (13). Tumor cells secrete several chemokines and chemokine receptors were identified on their surface at different levels of expression (14). According to current knowledge, the expression of specific chemokines and their receptors in tumor cells play a dual role in the oncogenic process (15). On the one hand, secreted chemokines by cancer-initiating cells and by normal surrounding cells may limit neoplastic progression by increasing leukocyte migration within the tumor, which eventually results in the induction of long-term antitumor immunity (15). Opposite to this process, other chemokines may facilitate tumor cell growth: (i) by recruiting endothelial cells; (ii) by subverting immunologic surveillance; and (iii) by maneuvering the tumor leukocyte profile, thus making feasible the escape from antitumor immune surveillance. More importantly, chemokines produced by tumor cells are believed to be involved in the metastatic process (15–17). Thus, tumor cells actively secrete and, owing to the presence of specific receptors on their cell membrane, respond to chemokines, which represent essential mediators influencing tumor progression (Figure 1).

## Thyroid Cancer

Thyroid cancer, the most prevalent endocrine malignancy, showed a sharp increase in its incidence in the last years, probably due to the routine use of neck ultrasound (18). Among all malignancies, the prevalence of thyroid cancer is relatively low (less than 1% of all malignant tumors in humans), but benign nodules are extremely frequent in the general population. Thus, the differential diagnosis between benign and malignant nodules is of great clinical relevance, to reduce the number of unnecessary thyroidectomies. Although the majority of differentiated thyroid cancers are slow growing and clinically indolent malignancies associated with an overall good prognosis (18), a recent survey reported a trend for increased mortality rate, mainly due to advanced-stage papillary thyroid cancer (PTC) (17). The availability of markers of thyroid cancer aggressiveness would allow the use of targeted therapies in selected cases, thus reducing the risk of over treating the vast majority of patients (18–20).

### Thyroid Cancer Microenvironment

Similar to other human malignancies, thyroid cancer microenvironment is composed of a mixture of immune cells (macrophages, mast cells, neutrophils, and lymphocytes) and soluble mediators (chemokines, cytokines, and growth factors), which are present within and nearby the primary thyroid tumor (21–23). Chemokines, which attract specific subpopulations of immune cell into the tumor site, are active immune mediators playing a role in thyroid tumor progression. Several *in vitro* and *in vivo* studies evaluated the role of chemokines in thyroid cancer and provided evidence for their ability to attract and maintain immune cells at the tumor site. Moreover, specific chemokines exert on thyroid cancer cells pro-tumorigenic actions, which include proangiogenetic, cytoproliferative, and pro-metastatic effects (24).

### Chemokines and Thyroid Cancer

Chemokines are a family of structurally related pro-inflammatory peptides of low-molecular weight, characterized by chemotactic activity. Originally, it was believed that the only function of chemokines was to recruit leukocytes to inflammatory sites. Subsequently, it was shown that chemokines also play a role in tumor cell growth, angiogenesis, and organ sclerosis. Currently, chemokines are classified in four families named C, CXC, CX3C, and CC according to the presence of a conserved amino-proximal cysteine residue in their NH2 terminal portion (23, 25, 26). With specific regard to thyroid cancer, a number of chemokines have been investigated for their antitumor or tumor-promoting activity.

### Chemokines With Antitumor Effects in Thyroid Cancer

#### CXC Motif Ligand 10

CXC motif ligand 10, previously known as interferon (IFN)-γ-induced protein 10 (IP-10), is secreted by several cell types (T lymphocytes, neutrophils, monocytes, endothelial cells, fibroblasts, keratinocytes, and pre-adipocytes), and also by normal thyroid cells (25, 27, 28). It binds the chemokine receptor CXCR3. CXCL10 is generally viewed as a mediator of host immune response, specifically T helper 1 cells, and its role in autoimmune endocrine diseases is well known (25, 27, 29, 30). Data derived from studies in other human malignancies support an antitumor effect for CXCL10, due to its anti-angiogenic properties (31). It was shown that CXCL10, being secreted within the tumor, attenuates the process of new vessels formation and leads to reduced tumor growth in non-small cell lung cancer (31, 32). A higher expression of CXCL10 was also related to an improved survival of patients with colon–rectal cancer (31, 33). In line with these observations, neutralization studies using anti-CXCL10 antibody demonstrated that this chemokine inhibits angiogenesis and chemotaxis in models of murine colon, mammary, and lung cancer (34). These antitumor effects of CXCL10 are supposed to be, at least in part, related to promotion of immune activities against tumor cells (31).

Although the antitumor effects of CXCL10 were not deeply investigated in thyroid cancer, currently available evidence indicates that: (i) oncogenic RET/PTC rearrangements and/or activating mutations of BRAF and RAS oncogenes activate a transcriptional program in PTC cells, which also involves an upregulation of CXCL10 (35); (ii) in thyroid PCCL3 cancer cells, RET/PTC rearrangements result in the early activation of genes being involved in the regulation of CXCL10 (36); and (iii) when compared with normal thyroid cells, PTC cells show a nearly 10-fold higher secretion of CXCL10 after stimulation with IFN-γ + TNF-α (37). The significance of the above findings in thyroid cancer remains to be elucidated, but data from other malignancies support the hypothesis that CXCL10 might have an antitumor effect.

### Chemokines With Tumor-Promoting Effects in Thyroid Cancer

Several chemokines are currently blamed for a tumor-promoting activity in thyroid cancer (Table 1).

**Table 1:** Chemokines with pro-tumorigenic effects in thyroid cancer microenvironment.

Family	Nomenclature (older names in brackets)	Receptors	Role in thyroid cancer	Reference
CC	*CCL15* (MIP-1 δ, LKN-1, HCC-2, MIP-5, NCC-3)	CCR1, CCR3	Recruits TAMs	(38)
CXC	*CXCL12* (SDF-1α/β)	CXCR4, CXCR7	Regulates neoplastic cell migration in PTC	(39, 40)
CXC	*CXCL16* (SR-PSOX)	CXCR6	Favors migration of neoplastic cells	(41)
CXC	*CXCL1* (GRO-α)	CXCR1, CXCR2	Favors proliferation, survival, and invasive behavior of thyroid cancer cells	(42)
CC	*CCL20* (LARC, MIP3α)	CCR6	Favors recruitment of DCs and neoplastic cell migration in PTC	(43–48)
CC	*CCL2* (MCP-1)	CCR2	Recruits TAMs	(49, 50)
CXC	*CXCL8* (IL-8, NAP-1)	CXCR1, CXCR2	Favors neutrophils recruitment, migration of neoplastic cells, EMT, and cell proliferation	(51–55)

*TAMs, tumor-associated macrophages; PTC, papillary thyroid cancer; DCs, dendritic cells; EMT, epithelial–mesenchymal transition; NAP-1, neutrophil attractant/activation protein-1; IL-8, interleukin-8; LARC, liver activation regulated chemokine; MIP3α, macrophage inflammatory protein-3; MCP-1, monocyte chemoattractant protein-1; CXCL12, C–X–C motif ligand 12*.

#### CCL15

CCL15, a potent chemoattractant for leukocytes and endothelial cells, binds the receptor CCR1 (56, 57). A recent *in vitro* study showed that CCL15 produced by follicular thyroid cancer cells is responsible for the recruitment of tumor-associated macrophages (TAMs) (38). TAMs are infiltrating inflammatory cells, which may comprise as much as 50% of the tumor mass (58). Their presence was found to be positively correlated with thyroid cancer cell growth and metastatic potential, and with a reduced patient’s survival (38, 51). The concentrations of CCL15 were reported to be significantly greater in follicular cancers when compared with follicular adenomas. This observation might imply that estimating the amount of TAMs (being attracted by CCL15) in follicular thyroid lesions would be clinically useful for the pre-surgery differentiation of follicular cancer from follicular adenoma (38).

#### CXC Motif Ligand 12

CXC motif ligand 12, also known as stromal cell-derived factor-1, binds to the CXCR4 (39) and to the CXCR7 receptors (59, 60). CXCL12 displays a strong chemotactic power toward lymphocytes, and it is known to play an important role in angiogenesis and tumor cell migration (39). The expression of CXCL12, CXCR4, and CXCR7 was detected in thyroid cancer tissue specimens (39, 61–63). Chung et al. demonstrated that, compared with non-cancerous thyroid lesions, CXCL12 is typically overexpressed in human PTC, regardless of its histologic subtypes. In this study, the sensitivity and specificity of using CXCL12 as a diagnostic marker for PTC was 90.8 and 96.8%, respectively (39). Immunohistochemistry studies also showed that 90% of PTC specimens were positive for CXCL12, whereas only 10.5% of follicular thyroid cancers showed positive staining for this chemokine (63). The absence of CXCL12 in non-cancerous thyroid lesions and in other thyroid malignancies, such as medullary and anaplastic thyroid cancers, supported the opinion that CXCL12 is specifically associated with PTC (40). In a subsequent study, the same authors provided evidence that the CXCL12–CXCR4/CXCR7 axis exerts its action in thyroid cancer cells by increasing their migration and invasiveness *via* Akt (also known as protein kinase B) and extracellular signal-regulated kinase signaling, and by activating matrix metalloproteinase-2 (40).

#### CXCL16

CXCL16 is a ligand for CXCR6 and signals through the Akt pathway (64). CXCL16 is abundantly present in the conditioned medium of macrophage–PTC cells co-cultures, being mainly produced by PTC-stimulated macrophages (41). Cho et al. demonstrated that an anti-CXCL16 neutralizing antibody, as well as an Akt inhibitor, attenuated the macrophage-dependent migration of PTC cells. This finding suggests that CXCL16 is involved in the macrophage-mediated actions promoting migration and invasiveness of PTC cells. In line with these data, the expression of CXCL16 and of another chemokine (CXCR6) was associated with the presence of lymph node metastasis in patients with PTC (41).

#### CXCL1

CXCL1, previously named GRO-α, is a growth factor that binds the CXCR2 receptor, and it is classically viewed as a chemoattractant for neutrophils. CXCL1 was found to be highly expressed in normal and pathologic thyroid tissue (65). An indirect role for CXCL1 in thyroid cancer progression was hypothesized in a study performed on mast cells (42). Mast cells are present in the tumor microenvironment where they influence angiogenesis and tumor invasion of the extracellular matrix by releasing cytokines and proteases (66–69). Mast cell-released mediators enhance proliferation, survival, and invasive behavior of thyroid cancer cells *in vitro*. Furthermore, mast cells promote the growth of thyroid carcinoma xenografts in nude mice (42). It is known that a complex mixture of mediators, contained in thyroid cancer cells conditioned medium, is responsible for mast cell activation. Although these mediators remain to be fully identified, Melillo et al. hypothesized that CXCL1 could mediate the proliferative effect of mast cell conditioned medium on thyroid carcinoma cell lines (42). Indeed, the addition of CXCL1 to the immune-depleted mast cell-conditioned medium rescued the proliferating effect of the complete medium.

#### CCL20

As a member of the CC-chemokine family, chemokine ligand 20 (CCL20), also named liver activation-regulated chemokine or macrophage inflammatory protein-3, is the exclusive ligand for the CCR6 receptor (70). The gene encoding for CCL20 belongs to the group of chemokine genes, which are highly expressed in PTC specimens compared with normal thyroid tissue (43). Its receptor (CCR6) was also found to be highly expressed in thyroid cancer cells (44). Immunohistochemical analysis demonstrated that CCL20 is strongly and diffusely expressed in tumor cells of the majority of well and poorly differentiated PTC. Only a minority of follicular carcinomas stained positive for CCL20, which was not detected in follicular adenoma or normal thyroid tissue (45). A subsequent flow cytometry study confirmed that CCR6 is expressed on a relevant proportion of TPC-1 (bearing the RET/PTC rearrangement) and BCPAP (bearing the BRAF mutation) tumor cells, but not in normal human thyroid cells in basal conditions. TNF-α induced a significant increase in the percentage of cells expressing CCR6, both in TPC-1 and BCPAP tumor cell lines, but not in normal human thyroid cells (46). TNF-α treatment increased the CCL20-promoted migration of TPC-1 and BCPAP tumor cells, but a similar effect was not observed in normal human thyroid cells (46). These actions of CCL20 resulted in the acquisition of a more aggressive behavior by the tumor. In agreement with these findings, previous observations indicated that the overexpression of CCR6 enhances the aggressiveness of colon–rectal cancer (71). The specificity of this CCL20–CCR6 interaction was demonstrated by the fact that CCR6 knockdown inhibits the CCL20-induced invasiveness and migration of thyroid cancer cells (47).

The role of CCL20–CCR6 interplay was further characterized by Scarpino et al. who provided evidence that this interaction is required for the migration of immature DCs in PTC (72). Indeed, CCR6 is exclusively expressed in human immature DC, which predominate in the immune infiltrate of PTC (45, 72, 73). These data indicate that in PTC the secretion of CCL20 by tumor cells promotes the migration of CCR6^+^ immature DC into the tumor. This notion is of particular relevance because the burden of DC infiltration was demonstrated to be closely linked to the prognosis of several malignant tumors (74–77) including, at least in some investigators’ view, thyroid cancer (78, 79).

#### CCL2

The identification of CCL2 (chemokine monocyte chemoattractant protein-1) and its receptor CCR2 (80) greatly contributed to the studies evaluating the mechanisms of monocyte trafficking during inflammatory responses and cancer development. The secretion of CCL2 has been demonstrated in several cancer cell types (7), but data in thyroid cancer are scanty. Tanaka et al. found a significant relationship between the expression of CCL2 in tumor cells and the presence of lymph node metastases in patients with PTC. Furthermore, higher expression levels of CCL2 characterized those patients with recurrent PTC as opposed to the successfully cured ones (49). The role of CCL2 in promoting TAM infiltration in PTC emerged after the finding that the oncogenic activation of BRAF in murine thyroids increased the expression of CCL2, which was rapidly followed by a robust recruitment of TAM expressing the CCR2 receptor. Furthermore, the selective targeting of CCR2-expressing cells during BRAF activation significantly reduced TAM density and retarded the development and progression of PTC. Targeting CCR2-positive cells in mice bearing an advanced PTC resulted in a reduced TAM density and in the regression of smaller, more differentiated, thyroid cancer foci containing fewer tall cells and poorly differentiated areas (50).

### CXCL8

At present, CXCL8 is probably the most studied chemokine in human cancer (81). Before addressing the link between CXCL8 and thyroid cancer, we will briefly review current evidence demonstrating the crucial role of CXCL8 in human malignancies of non-thyroid origin.

#### CXCL8 and Malignancies of Non-Thyroid Origin

CXCL8 is still often referred to as interleukin-8 or neutrophil attractant/activation protein-1 (52, 82). CXCL8 was first identified for its role in attracting neutrophils and other inflammatory cells, both to inflammation and tissue injury sites. The discovery of CXCL8 represented a landmark in immunology, because this chemokine was the first immune molecule found to have a chemoattractant activity on selected subpopulations of leukocytes (82, 83).

Neutrophils expressing CXCR1 and CXCR2 are attracted to the tumor site following a gradient of concentration of CXCL8 secreted by tumor cells. The presence of neutrophils in the tumor microenvironment is of great relevance. Indeed, once in site, neutrophils release enzymes, which are responsible for remodeling the extracellular matrix. This effect facilitate the migration of tumor cells and their entry in the vascular bed, thus allowing the travel to metastatic sites (84). However, the spectrum of CXCL8 actions is much wider. Besides acting as a chemoattractant, the binding of CXCL8 to its receptors (CXCR1 and CXCR2) results in the activation of CXCR1/CXCR2-expressing cells (85, 86). The expression of the CXCL8 gene and the subsequent secretion of the correspondent protein are primarily regulated by the transcription factors nuclear factor-κB (NF-κB) and activator protein-1 (AP1). However, experimental evidence suggests that other, yet to be unveiled, pathways are also involved (87). Owing to its potent pro-inflammatory properties, CXCL8 expression in normal tissues is low or undetectable (81), while an increased expression of CXCL8 was detected in several human solid tumors such as melanoma (88), squamous cell carcinoma (89), cervical (90), ovarian (91), non-small cell lung (92), colon (93), and gastric (94) cancers. Brain, breast, kidney, prostate, and thyroid cancers, as well as hematological malignancies (acute myelogenous leukemia, chronic lymphocytic leukemia, and Hodgkin’s lymphoma), also showed an increased expression of CXCL8 (81). The widespread expression of CXCL8 in human malignancies is of particular relevance, because the recently identified pleiotropic effects of this chemokine influence cell growth, angiogenesis, invasiveness, and metastatic potential.

Following its secretion by cancer cells, CXCL8 can enhance their proliferation rate and survival through autocrine signaling pathways. CXCL8 signaling triggers the epidermal growth factor receptor, thus promoting the downstream activation of the mitogen-activated protein kinase (MAPK) signaling *via* the monomeric small G protein, Ras-GTPase (95, 96). In several cancer cell lines, it was demonstrated that the activation of MAPK by CXCL8 results in increased cell proliferation and survival (93, 95–99). As shown in melanoma and in cancers of the liver, pancreas, and colon–rectum (100–103), CXCL8 functions as an autocrine growth factor.

The growth of solid tumors is dependent on the development of new blood vessels from preexisting capillaries, thus fulfilling the need of feeding cancer cells. Angiogenesis is a critical process in the development of malignancy and occurs in various stages of tumor progression. The formation of new blood vessels and lymphatics is a complex process characterized by multistep events, which require the intervention of proangiogenic factors, among which CXCL8 is a powerful one (104). The proangiogenic effect of CXCL8 was first demonstrated by Strieter et al. in the rabbit cornea (105) and further characterized in different types of cancer. The angiogenic effect of CXCL8 occurs after its binding to CXCR1 and CXCR2 receptors, which are expressed by endothelial cells (104). This binding results in endothelial cells activation, chemotaxis (as shown by *in vitro* experiments), and formation of new blood vessels *in vivo* (106, 107). This process is exemplified by the observation that in human gastrointestinal cancer (94) the concentrations of CXCL8 in tissues and in the circulation are correlated with the amount of new blood vessels within the tumor.

In xenografted and orthotopic *in vivo* models of cancer (86, 108–110), the metastatic potential of many solid tumors was found to be positively related with the secretion of CXCL8. The action of CXCL8 is mainly exerted through the recruitment of neutrophils (moving from blood vessels to the tumor site), which enable the migration of tumor cells. Thus, it is currently accepted that CXCL8 has pro-metastatic properties. Further support to this statement stems from the demonstration that CXCL8 is strongly involved in the epithelial–mesenchymal transition (EMT), a complex molecular program, whereby epithelial cells lose their typical features (i.e., apical–basolateral polarity, extensive intercellular adhesions, and contact growth inhibition) in favor of acquiring mesenchymal features, which include edge trailing, edge asymmetry, and loss of intercellular contact. Motility and invasiveness are also increased (111, 112). These CXCL8-induced changes were characterized in cells deriving from cancers of the colon (113), of the nasopharynx (114), and of the breast (115). The tumor-promoting effect of CXCL8 is further strengthened by the observation that, while this chemokine promotes EMT, EMT by itself promotes CXCL8 secretion, thus producing a loop-reinforcing cascade, which was demonstrated in colon and breast cancer cells (116, 117). Several mechanisms are likely to be involved in this process: (i) the aberrant nuclear localization of the tight junction protein ZO-1, which, being associated with EMT, leads to enhanced CXCL8 expression (118); (ii) the overexpression of Snail, a zinc-finger transcriptional repressor tightly controlling EMT (119), which directly activates the transcription of CXCL8 by binding E-boxes within the CXCL8 gene promoter (113, 120). A summary of the multifaceted pro tumorigenic effects of CXCL8 in cancer is given in Table 2.

**Table 2:** Multifaceted tumor-promoting actions of CXCL8.

CXCL8 effects	Evaluation	Type of cancer cells	Reference
Tumor cell growth	*In vitro*	Liver, pancreas, colon, colon–rectum, gastric, ovarian, non-small lung, prostate	(93, 95–102)
Angiogenesis	*In vitro in vivo*	Breast, gastric, gastrointestinal	(101, 104–108)
Induction of EMT and/or stemness	*In vitro in vivo*	Colon, nasopharyngeal, breast, colon, non-small lung, thyroid	(87, 109–119)
Formation of spheres, self-renewal of stem cells, and tumor-initiating ability	*In vitro*	Thyroid cancer stem cells	(55)
Induction of neoplastic cell migration	*In vitro*	Thyroid cells TPC-1 and BCPAP	(121)
Increase of metastatic spread	*In vivo*	Bladder, thyroid	(51, 87, 109, 110)

*TPC-1, papillary thyroid cancer cell line with RET/PTC rearrangement; BCPAP, papillary thyroid cancer cell line with BRAFV600e mutation; EMT, epithelial–mesenchymal transition*.

Based on the multifaceted tumor-promoting effects of CXCL8, the possibility to use this chemokine as a tumor biomarker appeared a fascinating perspective. With this aim, several studies measured the concentrations of CXCL8 in the serum and in tissues of cancer patients. The results of these studies indicated that the concentrations of CXCL8 are higher in cancer patients compared with healthy controls. In several types of cancer including melanoma, renal cell carcinoma, non-small cell lung cancer, and hepatocellular carcinoma, the levels of CXCL8, as measured in serum and in tissues, correlate with tumor size, depth of infiltration, stage, and prognosis (122). Moreover, Broutin et al. found significantly higher circulating levels of CXCL8 in metastatic medullary thyroid cancer patients as opposed to healthy donors (123). As far as DTC is concerned limited studies are available. However, two independent studies reported higher circulating concentrations of CXCL8 in patients with DTC as opposed to patients with benign nodules (124, 125). From a clinical point of view, the main message stemming from these results is that lower serum levels of CXCL8 characterize a less aggressive course of malignancy and a better response to anticancer therapy (122).

#### CXCL8 and Thyroid Cancer

At present, CXCL8, in view of its proven pro-tumorigenic effects, is the most extensively investigated chemokine involved in thyroid cancer. In this section, we will review currently available data regarding: (i) the ability of both normal and tumor thyroid cells to secrete CXCL8; (ii) the direct/indirect pro-tumorigenic effects of CXCL8, as shown by *in vitro* and *in vivo* studies in thyroid cancer cells; and (iii) the pharmacologic strategies for lowering CXCL8 secretion and/or for targeting its effects in thyroid cancer.

##### Secretion of CXCL8 by Thyroid Cells

The link between CXCL8 and the thyroid was discovered in the far 1992, when the first demonstration was provided that normal thyroid cells in primary culture secrete CXCL8 (126). More recent studies demonstrated that iodide positively regulates the expression of the CXCL8 gene (127) and that TSH induces the production of CXCL8 in Graves’ disease derived peripheral blood fibrocytes expressing the TSH receptor. These findings suggested that CXCL8 is somehow linked to thyroid homeostasis (128).

Initially, CXCL8 was hypothesized to play a role in the development of autoimmune thyroid diseases (129). More recently, the involvement of CXCL8 in thyroid oncogenesis emerged as a new frontier of research (129). The first demonstration that thyroid cancer cells secrete CXCL8 was provided by Yoshida et al., who reported that this chemokine was secreted by KHM-5M cells obtained from an undifferentiated thyroid cancer (53). The presence of a neutrophil infiltrate within the tumor of nude mice transplanted with KHM-5M cancer cells strongly supported a role for CXCL8 produced by neoplastic thyroid cells in the recruitment of neutrophils at the tumor site. Subsequently, it was found that, besides KHM-5M cells, neoplastic cells derived from other types of thyroid cancer secrete CXCL8, which is involved in thyroid cancer-related inflammation. In 2002, Basolo et al. showed that CXCL8 is the most abundant chemokine being secreted in cell culture supernatants of papillary, anaplastic, and poorly differentiated thyroid carcinoma cell lines (130). In agreement with these findings, Borrello et al. demonstrated that the pro-inflammatory program elicited by RET-PTC rearrangement stimulates a panel of chemokine genes among which the CXCL8 gene is the main upregulated one. As a consequence, the correspondent protein is the main secretory product (131). Subsequently, Muzza et al. found a significantly higher expression of the CXCL8 gene in PTC specimens (43). More importantly, the CXCL8 gene was found to be similarly expressed in non-neoplastic tissue specimens of thyroiditis and in normal thyroid tissue. This observation, besides being an indirect confirmation that CXCL8 has a minor if any role in autoimmune thyroid diseases, strongly supports the concept that the cytokine/chemokine milieu sustaining chronic autoimmune thyroiditis is different from that involved in thyroid tumor-related inflammation (43, 54, 129).

The modality of CXCL8 secretion by thyroid cancer cells was investigated in several studies. Currently, available evidence indicates that TPC-1 (RET/PTC1 mutated) and BCPAP (BRAF-V600E mutated) thyroid tumor cells secrete significantly different amounts of CXCL8 as measured in the cell culture supernatants, both basally and after TNF-α stimulation (132). BCPAP cells displayed a higher basal secretion of CXCL8, but their response to TNF-α was smaller than that of TPC-1 cells (132). The main concept emerging from these *in vitro* data is that the type of oncogenic lesion harbored by thyroid cancer cells accounts for their different ability to secrete CXCL8. In line with this interpretation, Zhang et al. showed that, among PTC cells, those overexpressing the chemokine receptor CXCR7 (the receptor for CXCL12) secrete higher amounts of CXCL8, which reverts following CXCR7 inhibition (133). Going deeper in understanding the mechanisms responsible for CXCL8 secretion is a complicated issue, because CXCL8 secretion can be induced both by exogenous and by endogenous (at the DNA level) factors. Accordingly, a number of different stimuli, including inflammatory signals (e.g., TNF-α and IL-1β), chemical and environmental factors (e.g., chemotherapy agents and hypoxia), and steroid hormones (e.g., androgens, estrogens, and dexamethasone), all contribute in regulating the expression of CXCL8 (86, 134).

##### Pro-Tumorigenic Effects of CXCL8 in Thyroid Cancer

While solid evidence already indicated that thyroid cancer cells secrete CXCL8, the role of this chemokine in influencing the biological behavior of thyroid cancer and more specifically its pro-tumorigenic effect had to be established. At present, a widely addressed topic regards the ability of CXCL8 to promote the metastatic spread of thyroid cancer cells. In line with what reported in other human malignancies, Visciano et al. elegantly demonstrated that CXCL8 is the main mediator of stemness and of EMT in thyroid cancer cells (55). Incubation of mast cells with thyroid cancer cell-derived conditioned medium produced a strong upregulation of a set of selected cytokines (TNF-α, CXCL6, and CXCL8), which consistently induced EMT in thyroid cancer cells. Immune depletion experiments not only identified CXCL8 as the most powerful inducer of EMT but also provided evidence that the presence of CXCL8 is a necessary condition for inducing EMT in thyroid cancer cells. The authors also demonstrated that this effect would be exerted through an Akt–Slug-dependent pathway (55). In particular, the *in vitro* inhibition of Akt (a downstream mediator of CXCL8 action), prevented Slug accumulation (an important mediator of EMT and Stemness), eventually blocking EMT and stemness of thyroid cancer cells. Thus, CXCL8 signaling appears to be a crucial step in this oncogenic pathway (55). The same group of investigators showed that CXCL8, through an autocrine circuit mediated by the CXCR1 receptor, is involved in the formation of spheres, in their self-renewal and in the tumor-initiating ability of thyroid cancer stem cells. The identification of this autocrine pro-tumorigenic action of CXCL8 is of great relevance, because it clearly indicates that targeting the CXCL8–CXCR1 circuit could produce therapeutic benefits, at least in patients bearing an aggressive radioiodine-refractory thyroid cancer (135). In line with the above reported findings, it was shown that the incubation of TPC-1 and BCPAP thyroid cancer cell lines with recombinant human (rh)-CXCL8, significantly increased cell migration, as assessed by an *in vitro* migration assays (121).

**Figure 2:**
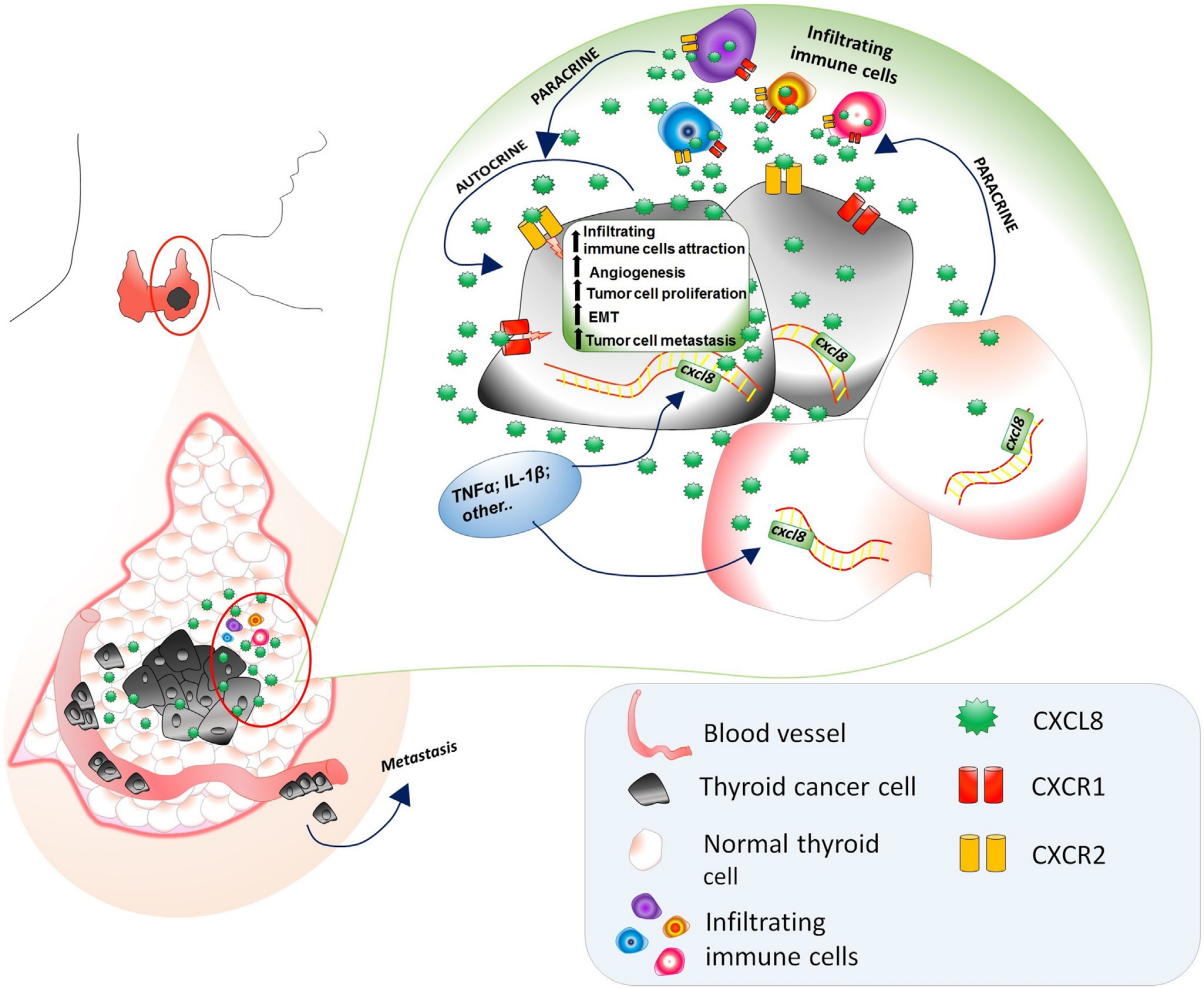
Multifaceted pro-tumorigenic effects of CXCL8 in thyroid cancer microenvironment. CXCL8, being produced by both normal and tumor thyroid cells, exerts a chemotactic action on circulating immune cells (expressing CXCR1 and CXCR2), which further secrete CXCL8. The presence of TNFα, Ilβ, and other CXCL8 stimulating factors in the thyroid cancer microenvironments induces an upregulation of the CXCL8 secretion by both normal and cancer thyroid cells. The expression of CXCR1 and CXCR2 on thyroid tumor cells accounts for the autocrine/paracrine signaling exerted by CXCL8 secreted by normal thyroid cells, thyroid cancer cells themselves, and infiltrating immune cells resulting in an increase of (i) recruitment of infiltrating immune cells, (ii) angiogenesis, (iii) tumor cell proliferation, (iv) EMT, and (v) tumor cell migration in the vascular bed and their journey to metastatic sites.

Moving to *in vivo* experiences, the involvement of CXCL8 in the metastatic process of thyroid cancer cells was studied in NOD/SCID mice transfected with a thyroid cancer cell line. Treatment with rh-CXCL8 significantly increased the metastatic spread, ultimately resulting in an increased mortality. These data unambiguously proved that the administration of exogenous CXCL8 has a powerful pro-metastatic role, at least in this animal model of thyroid cancer (51). It was hypothesized that CXCL8 would exert its active role in thyroid cancer progression by paracrine binding to CXCR1 and CXCR2 expressed on the surface of PTC cells (51). A comprehensive scheme of the multifaceted pro-tumorigenic effects of CXCL8 in thyroid cancer is given in Figure 2.

In line with *in vitro* findings and data from animal studies, Bauerle et al. reported that patients with high-risk thyroid cancer display significantly higher intra-tumor levels of CXCL8 mRNA than those found in patients with low-risk disease (136). These data give indirect support to the existence of a positive relation between higher intra-tumor CXCL8 levels and a more aggressive course of thyroid cancer. The only study in which the circulating levels of CXCL8 were measured in patients with PTC reported significantly higher concentrations of CXCL8 in affected patients when compared with healthy controls (137). Although these results derive from a single study, they are in line with those performed in patients affected by other types of cancer. Taken together, *in vitro* and *in vivo* data provide evidence for an action of CXCL8 as tumor-promoting agent and indicate that lowering CXCL8 levels in thyroid cancer microenvironment could be of therapeutic benefits.

#### Strategies for Reducing CXCL8 Effects in Cancers of Non-Thyroid Origin

##### Targeting CXCL8

Moving from the idea that lowering the levels of CXCL8 would result in the attenuation of its pro-tumorigenic effects, several attempts were done to reduce CXCL8 production by cancer cells or to block its effects. The neutralization of CXCL8 *via* ABX-IL8 (a blocking antibody) significantly reduced neoplastic cell invasion and angiogenesis in mice bearing a melanoma (138). Similarly, in a mice model of malignant mesothelioma, targeting CXCL8 by a neutralizing monoclonal antibody (mAb) decreased tumor progression (139). Subsequently, different agents, ranging from chemotherapeutic drugs to pharmacologic or natural compounds with anti-inflammatory properties, were tested for their ability to inhibit CXCL8 production (Table 3).

**Table 3:** CXL8-lowering agents tested in several types of cancer cells.

Compound	Type of cancer cells	Consequent anticancer effect	Reference
BRAF inhibitors	Melanoma cells	Anti-proliferative and anti-metastatic	(140)
Dehydroepiandrosterone	Breast cancer cells	Anti-proliferative	(141)
*Rhus coriaria*	Breast cancer cells	Anti-proliferative and anti-metastatic	(142)
Metformin	Endometrial stromal cells, thyroid cells	Not assessed	(143)
1α,25-dihydroxyvitamin D3	Prostate cancer cells	Anti-angiogenic	(144)
Sunitinib	Medullary thyroid cancer cells	Anti-angiogenic, anti-proliferative	(123)
Interferon-γ	Thyroid cancer cells	Anti-metastatic	(121)
Oncolytic adenovirus dl922-947	Anaplastic thyroid cancer	Anti-angiogenic, inhibition of macrophage infiltration	(145)

##### Lowering CXCL8

The results obtained with CXCL8 neutralizing mAb were consistently confirmed by studies aimed at lowering the secretion of CXCL8 by cancer cells. In human melanoma xenografts, a BRAF inhibitor, with anti-proliferative and anti-metastatic properties, inhibited CXCL8 synthesis (140). The administration of dehydroepiandrosterone, an adrenal hormone with a protective role against cancer, inhibited the secretion of several chemokines including CXCL8, which was paralleled by a reduction of breast cancer cells growth (141).

*In vitro* experiments also showed that *Rhus coriaria* (a fruit displaying anticancer effects) dose dependently inhibits CXCL8 secretion in breast cancer cells; thus accounting, at least in part, for the inhibition of tumor growth and metastasis mediated by *R. coriaria* (142).

Metformin, an oral hypoglycemic agent traditionally used for the treatment of type 2 diabetes mellitus, was recently identified as an antitumor agent (146), which negatively modulates the secretion of CXCL8 in endometrial stromal cells (143). Similarly, vitamin 1α,25-OH-D3 was found to suppress CXCL8-mediated angiogenesis in prostate cancer cells (144). Sunitinib, a multi-kinase inhibitor with both anti-angiogenic and antitumor activities, was demonstrated to strongly reduce CXCL8 secretion in medullary thyroid cancer cells (123). More importantly, CXCL8 was identified as a potential biomarker of sunitinib responsiveness, because *in vivo* the circulating levels of CXCL8 dramatically decreased following sunitinib administration in medullary thyroid cancer xenografts (123). Taken together, these data strongly support the idea that CXCL8 might serve both as a potential therapeutic target and a clinical biomarker of some types of cancer. Therapeutic strategies aimed at lowering or targeting CXCL8 represent a promising perspective for future antitumor therapies.

#### Strategies for Reducing CXCL8 Effects in Thyroid Cancer

##### Targeting CXCL8

The first proof that targeting CXCL8 within the tumor microenvironment could produce therapeutic benefits was provided by Fang et al. in the previously mentioned study performed in NOD/SCID mice harboring thyroid cancer (51). Indeed, while the administration of rh-CXCL8 promoted tumor spread and reduced mice survival, targeting CXCL8 by a neutralizing Ab dose dependently reduced the metastatic spread of thyroid cancer cells and prolonged mice survival (51). More recently, Liotti et al. provided evidence that reparixin (an inhibitor of the CXCL8 receptors, CXCR1, and CXCR2) reduces cell survival, proliferation, EMT, and stemness of several thyroid cancer cell lines (147). The specificity of this finding was demonstrated by the lack of any effect of reparaxin on viability of normal thyrocytes, which do not express CXCR1 and CXCR2 and by the fact that silencing of CXCR1 and CXCR2 abolished these effects.

Furthermore, reparixin significantly inhibited thyroid cancer cell tumorigenicity in immunodeficient mice. While potentiating of several chemotherapies on thyroid cancer cell xenotransplants in mice (147). Taken together, the above *in vitro* and *in vivo* findings would support the concept that targeting of CXCL8 receptors could represent a potential therapeutic strategy at least for aggressive DTC.

##### Lowering CXCL8

Several attempts were done to reduce the secretion of CXCL8 in the thyroid tumor microenvironment. Among the tested agents, metformin recently received attention. *In vitro ex vivo* studies demonstrated that metformin directly inhibits the growth of cancer cells derived from papillary and follicular thyroid cancer specimens (148, 149), being this effect exerted *via* an AMPK-mediated inhibition of the mTOR signaling pathway (148). An additional indirect antitumor effect of metformin was demonstrated in a subsequent *in vitro ex vivo* study (150). Indeed, metformin significantly and dose dependently reduced the secretion of CXCL8 induced by TNF-α in primary cultures of human thyroid cells obtained both from normal thyroid tissue and PTC surgical specimens. The same was not true in the thyroid tumor cell lines TPC-1 and BCPAP (150). At difference with metformin, AICAR (another AMPK activator with known anticancer properties) was recently demonstrated to inhibit CXCL8 secretion and cell migration in the thyroid tumor cell lines TPC-1 and BCPAP and in normal human thyroid cells (151). In line with the notion that CXCL8 promotes EMT (a crucial step in metastatization), the inhibition of cell migration by AICAR was observed only in those cells in which AICAR also inhibited CXCL8 secretion. These data would suggest that at least some of the anticancer effects of AICAR and inhibition of cell migration, in particular, would be exerted through the reduction of the CXCL8 levels.

Similarly, designed studies showed that IFN-γ does reduce the basal and the TNFα-stimulated secretion of CXCL8 both in normal thyroid cells and in BCPAP cells, but not in the TPC-1 thyroid cancer cell line (121, 152). The specificity of IFN-γ as a CXCL8-lowering agent in BCPAP cells was further characterized by a migration assay performed both in basal conditions and after stimulation with the rh-CXCL8 protein. In these experiments, rh-CXCL8 increased cell migration in both TPC-1 and BCPAP cells, but this effect was reverted by IFN-γ only in BCPAP cells (the ones in which IFN-γ also reduced CXCL8 secretion) (129). Taken together, the above results suggest that the pathways responsible for the secretion of CXCL8 differ in BCPAP cells, bearing the BRAF-V600E mutation, when compared with TPC-1 cells, bearing the RET/PTC rearrangement. This conclusion would fit with notions in human melanoma cells, which also bear the BRAF-V600E mutation and respond to a BRAF inhibitor with a reduced synthesis of CXCL8, thus resulting in anti-proliferative and anti-metastatic effects (140).

Additional evidences supporting the beneficial effects of lowering CXCL8 were provided also in anaplastic thyroid cancer cells. Indeed, it was recently reported that the oncolytic adenovirus dl922-947 significantly lowered the synthesis of CXCL8 (and of CCL2 as well) by displacing the transcription factor NF-κB p65 from its specific promoters. The reduction of CXCL8 (and CCL2) was paralleled by impaired tumor angiogenesis and decreased macrophage density both in the *in vitro* and *in vivo* models of mice anaplastic thyroid cancer xenograft (145). Similarly, camptothecin, an inhibitor of DNA topoisomerase-I, besides decreasing anaplastic thyroid cell proliferation, apoptosis, adhesion, and migration, it also lowers CXCL8 secretion *in vitro*. The inhibition of CXCL8 secretion by this compound would account for the decrease of tumor neoangiogenesis and vascularization *in vivo* (153).

At present, several agents, although with different strength of inhibition, turned out to be effective in reducing the secretion of CXCL8. However, none of them, at any concentration, was able to completely abolish the secretion of CXCL8. Failure to completely abolish CXCL8 secretion in the tumor microenvironment appears in line with the notion that CXCL8 secretion results from multiple intracellular signals and/or pathways, which appear to be different when normal or tumor cells are taken into account. In addition, among tumor cells, specific genetic lesions were found to be major determinants of the secretion of CXCL8 (86, 154). Initially, it was believed that the expression of CXCL8 mRNA in different thyroid cancer cell lines was mainly regulated by NF-κB. However, the fact that NF-κB interfering agents successfully inhibited CXCL8 secretion in some, but not all thyroid cell lines, suggested that multiple pathways are responsible for the secretion of CXCL8. These include, in adjunction to NF-κB signaling, the AP1, the hypoxia-inducible factor 1α, and the MAPK pathways (136, 150, 155).

## Conclusive Remarks

The inflammatory tumor microenvironment consists of a mixture of immune cells and soluble mediators. In the latter group, chemokines represent key factors for tumor initiation, maintenance, and progression. Chemokines are a group of immune molecules secreted by both tumor and normal surrounding cells. At present, CCL2, CCL15, CCL20, CXCL1, CXCL8, CXCL12, and CXCL16 are the main chemokines showing a role in thyroid cancer. Among them, CXCL8, previously demonstrated to exert several tumor-promoting effects in many human cancers, represents the most deeply investigated chemokine in thyroid tumor microenvironment. Differentiated thyroid cancers are the most prevalent endocrine malignancies being in most cases slow growing, clinically indolent lesions with an overall good prognosis and a very low-mortality rate. The availability of a marker of aggressiveness in the minority of patients harboring aggressive thyroid cancer would be welcome, because it would allow targeted therapies, while reducing the risk of over treating the vast majority of patients. Solid evidence from *in vitro* and *in vivo* studies supports a major role for CXCL8 in determining a more aggressive clinical course of differentiated thyroid cancer. CXCL8 favors tumor progression through several mechanisms, which include the promotion of thyroid cancer metastatic spread and EMT (Figure 2). Targeting of CXCL8 in thyroid tumors provides therapeutic benefits in experimental models. Thus, several agents were tested for their efficacy in inhibiting the secretion of CXCL8 in the thyroid cancer microenvironment. Inhibiting CXCL8 secretion turned out to be more difficult than it could be envisaged, even if several compounds allowed a certain degree of inhibition of CXCL8 secretion by thyroid tumor cells. Given the crucial importance of tumor-related inflammatory microenvironment in determining tumor progression, it appears likely that research to address the issue of lowering CXCL8 secretion will continue in the future.

## Author Contributions

All the authors contributed equally to the study.

## Conflict of Interest Statement

The authors declare that the research was conducted in the absence of any commercial or financial relationships that could be construed as a potential conflict of interest. The handling Editor declared a shared affiliation, though no other collaboration, with one of the authors, FL and with the reviewer, SF.
